# Alone and under pressure: the transdiagnostic role of loneliness, stress, and psychological inflexibility in university students

**DOI:** 10.3389/fpubh.2025.1642529

**Published:** 2025-08-14

**Authors:** Silvia L. Vaca-Gallegos, Mateo Peñaherrera-Aguirre, Daniela Batallas, Belén M. Paladines-Costa, Carla López-Nuñez, Pablo Ruisoto

**Affiliations:** ^1^Department of Psychology, Universidad Técnica Particular de Loja, Loja, Ecuador; ^2^School of Animal and Comparative Biomedical Sciences, University of Arizona, Tucson, AZ, United States; ^3^Laboratory of Social Cognitive Neuroscience, Department of Psychobiology and IDOCAL, University of Valencia, Valencia, Spain; ^4^Department of Personality, Assessment and Psychological Treatments, School of Psychology, University of Seville, Seville, Spain; ^5^Department of Health Sciences, Public University of Navarre, Pamplona, Spain; ^6^Navarra Institute for Health Research (IdiSNA), Pamplona, Spain; ^7^Institute for Advanced Social Research (I-COMMUNITAS), Pamplona, Spain

**Keywords:** loneliness, perceived stress, psychological inflexibility, mental health, life satisfaction, transdiagnostic model, public mental health

## Abstract

**Introduction:**

University students face growingmental health challenges that demand both clinical and population-level strategies. Psychological inflexibility, perceived stress, and loneliness have been proposed as key transdiagnostic factors influencing mental health, yet their interrelationships remain unclear.

**Methods:**

We conducted a cross-sectional survey among 7,905 students from 11 Ecuadorian universities. Validated instruments were used to assess psychological inflexibility (AAQ-II), perceived stress (PSS), loneliness (UCLA-3), anxiety and depression (PHQ-4), and life satisfaction (LSQ). Data were analysed using Sequential Canonical Analysis to examine direct and indirect associations among predictors and outcomes.

**Results:**

Analysis revealed a structured cascade: psychological inflexibility predicted perceived stress, which in turn predicted loneliness. All three variables contributed independently to mental health outcomes. Loneliness was the strongest predictor of anxiety, depression, and reduced life satisfaction, supporting its role as a chronic social stressor. Together, these factors explained 45% of the variance in a higher-order mental health factor and 35% of the variance in life satisfaction.

**Discussion:**

Findings underscore the need for integrated strategies in higher education that address both individual vulnerability and social isolation. Framed within Rose’s distinction between the causes of individual cases and the causes of population incidence, results highlight loneliness as a central target for preventive and clinical interventions.

## 1 Introduction

Mental health conditions are among the leading contributors to global disability (1), particularly among young adults. University students face unique developmental challenges—including academic demands, social transitions, and financial stressors—that heighten their vulnerability to psychological distress (2–4). In Ecuador, these vulnerabilities have been further intensified by the social and economic impact of the COVID-19 pandemic (5, 6). Despite this, research on student mental health in the region remains limited and fragmented (7, 8).

A growing body of literature supports the use of transdiagnostic frameworks to explain common mechanisms underlying diverse mental health outcomes (9). Rather than focusing on disorder-specific predictors, transdiagnostic models identify general psychological processes that contribute to multiple conditions—particularly anxiety, depression, and low life satisfaction. Among these, three constructs have gained considerable empirical support: psychological inflexibility, perceived stress, and loneliness.

Psychological inflexibility—the tendency to rigidly avoid or suppress unpleasant internal experiences—has been identified as a key predictor of psychological distress (10, 33). During the COVID-19 pandemic, higher levels of flexibility were associated with better emotional regulation and lower symptomatology (11, 12). Perceived psychological stress, shaped by one’s evaluation of environmental demands and coping resources, plays a central role in the onset and maintenance of mental health problems in university populations (13, 14). Loneliness, defined as the subjective experience of social disconnection (15), has also emerged as a powerful correlate of depression, anxiety, and suicide risk (30).

These variables likely interact in complex ways. Psychological inflexibility may amplify stress perception, while loneliness may act as a chronic social stressor. As Rose (34) famously argued, public health must distinguish between the causes of individual cases and the causes of incidence at the population level. In this context, inflexibility reflects individual vulnerability, whereas loneliness and chronic stress may represent contextual exposures that elevate incidence rates of poor mental health. Addressing these factors could therefore enhance both clinical outcomes and population-level mental health.

This study aimed to examine the interrelationships among psychological inflexibility, perceived stress, and loneliness, and their collective impact on anxiety, depression, and life satisfaction in a large sample of Ecuadorian university students. Using Sequential Canonical Analysis (SEQCA), we sought to identify direct and indirect pathways of influence to inform more effective, scalable interventions in higher education settings.

## 2 Materials and methods

### 2.1 Participants

A total of 7,905 university students from 11 higher education institutions in Ecuador participated in this cross-sectional study. Recruitment was conducted via institutional email invitations. Eligible participants were required to be enrolled for at least one full academic year and to complete the full survey. The average response rate was 47.8%, ranging from 39.1 to 56.3% across universities. The mean age of participants was 21.49 years (SD = 3.68); 46.26% were male (*n* = 3,656; M = 21.8, SD = 3.7), and 53.75% were female (*n* = 4,249; M = 21.2, SD = 3.7). All participants provided informed consent prior to their inclusion.

### 2.2 Measures

#### 2.2.1 Psychological inflexibility

Assessed with the Acceptance and Action Questionnaire-II (AAQ-II) (16), a 7-item instrument measuring experiential avoidance and cognitive rigidity. Responses range from 1 (never) to 7 (always), with higher scores indicating greater inflexibility. Cronbach’s alpha in this study was 0.919.

#### 2.2.2 Perceived stress

Measured using the 14-item Perceived Stress Scale (PSS) (13, 17). Items are rated from 0 (never) to 4 (very often), with higher scores reflecting higher perceived stress. Cronbach’s alpha was 0.768.

#### 2.2.3 Loneliness

Assessed using the 3-item UCLA Loneliness Scale—Short Form (18). Items are rated from 1 (hardly ever) to 3 (often), with higher scores indicating greater loneliness. Cronbach’s alpha was 0.844.

#### 2.2.4 Depressive and anxiety symptomatology

Evaluated via the Patient Health Questionnaire-4 (PHQ-4) (19), which includes two items for depression and two for anxiety. Total scores range from 0 to 12, with higher scores indicating greater psychological distress. Cronbach’s alpha was 0.808.

#### 2.2.5 Life satisfaction

Assessed through a single-item Satisfaction with Life Question (LSQ): “How satisfied are you with your life as a whole nowadays?,” rated on a scale from 0 (not at all) to 10 (completely) (20).

### 2.3 Design and procedure

A cross-sectional observational study was conducted. Data were collected using an online survey distributed via institutional emails from 11 Ecuadorian universities. Participation was anonymous and voluntary. To encourage honest responses, participants were assured confidentiality and received a brief individualized feedback report upon completion.

The study was approved by the Ethics Committee for Research Involving Human Beings (Comité de Ética de Investigación en Seres Humanos, CEISH) of the Ministry of Public Health of Ecuador (Approval code: MSP-DIS-2015-0088-O), and adhered to the principles of the Declaration of Helsinki (2008 revision). All participants provided written informed consent before enrollment.

### 2.4 Statistical analyses

We performed all statistical analyses using SAS 9.4 (35), the *psych* package (21) in R v 4.3.1, and UniMult 2.0 (22). Prior to the main analyses, all scale items were standardized using the PROC STANDARD function in SAS to ensure metric comparability across measures.

We first assessed the internal consistency of each instrument using Cronbach’s *α* and McDonald’s *ω* coefficients. To explore the latent structure of the scales, we applied two complementary procedures: unit-weighted factor (UWF) estimation and Principal Axis Factoring (PAF). UWF scores were calculated as the average of standardized item responses, yielding a composite indicator for each construct. PAF was used to extract latent dimensions, determine eigenvalues, and estimate the proportion of explained variance. As unit-weighted factor scores are computed following a mathematical procedure different from PFA-related scoring approaches (regression-based methods), it was pertinent to examine the degree of correspondence between UWF and the PAF factor structures via congruence coefficients (CCs), wherein a high degree of congruence indicates that the observed structure is not a mathematical artifact of either the extraction or the scoring procedures.

Following these analyses, we conducted a Sequential Canonical Analysis (SEQCA) to examine both direct and indirect relationships among the key variables. SEQCA is a multivariate modeling technique that uses a series of hierarchical linear regressions to estimate chains of influence, where outcome variables at each step become predictors in subsequent steps. This approach allows for the assessment of cumulative effects across conceptually ordered variables. The analytic sequence was structured as follows: Step 1: Psychological inflexibility (AAQ-II), sex, age, and region. Step 2: Perceived stress (PSS). Step 3: Loneliness (UCLA-3). Step 4: Mental health outcomes, modeled as a higher-order latent factor comprising depression, anxiety (PHQ-4), and reversed life satisfaction (LSQ). The present study employed PAFs and SEQCAs instead of CFAs and SEMs, as the sequential influence of the aforementioned variables had not been previously examined in this population. Future statistical examinations should confirm whether the results described in the present study replicate under confirmatory procedures using a sample collected from a similar population.

## 3 Results

### 3.1 Internal consistency and factor structure

All psychometric instruments demonstrated acceptable to excellent internal consistency. Cronbach’s alpha values were as follows: Acceptance and Action Questionnaire-II (AAQ-II) = 0.92, Perceived Stress Scale (PSS) = 0.77, UCLA Loneliness Scale = 0.85, and Patient Health Questionnaire-4 (PHQ-4) = 0.85. Subscale reliabilities for PHQ-4 were 0.81 for anxiety and 0.74 for depression. In terms of the reliability estimates based on McDonald’s *ω* the analysis revealed that the AAQ-II exhibited an adequate level of internal consistency (*ω* = 0.92). Similarly, the PSS featured an adequate level of internal consistency (*ω* = 0.78). The UCLA also displayed an adequate degree of internal consistency (ω = 0.85). Finally, the PHQ-4 featured an adequate degree of internal consistency (ω = 0.87).

The PSS exhibited a unidimensional structure, with Principal Axis Factor (PAF) loadings ranging from 0.149 to 0.703 and a PAF eigenvalue of 4.360. Unit-weighted factor (UWF) loadings ranged from 0.293 to 0.725. The congruence coefficient (CC) between UWF and PAF was 0.993, indicating high structural similarity (Table 1).

**Table 1:** Unit-weighted and principal axis factor loadings of the perceived stress scale (PSS).

Perceived stress scale (PSS) indicators	UWF loading	PAF loading
In the last month, how often have you been upset because of something that happened unexpectedly?	0.609	0.540
In the last month, how often have you felt that you were unable to control the important things in your life?	0.725	0.703
In the last month, how often have you felt nervous and “stressed”?	0.652	0.585
In the last month, how often have you dealt successfully with irritating life hassles?	0.426	0.391
In the last month, how often have you felt that you were effectively coping with important changes were occurring in your life?	0.497	0.492
In the last month, how often have you felt confident about your ability to handle your personal problems? (R)	0.493	0.641
In the last month, how often have you felt that things were going your way?	0.653	0.658
In the last month, how often have you found that you could not cope with all the things that you had to do?	0.582	0.501
In the last month, how often have you been able to control irritations in your life?	0.608	0.621
In the last month, how often have you felt that you were on top of things?	0.626	0.620
In the last month, how often have you been angered because of things that happened that were outside of your control?	0.642	0.568
In the last month, how often have you found yourself thinking about things that you have to accomplish?	0.293	0.149
In the last month, how often have you been able to control the way you spend your time?	0.445	0.400
In the last month, how often have you felt difficulties were piling up so high that you could not overcome them?	0.720	0.684

R: Reversed scored item.

The AAQ-II also showed a clear single-factor structure, with PAF loadings between 0.744 and 0.826, UWF loadings from 0.791 to 0.847, and an eigenvalue of 4.326. The congruence coefficient was 0.999 (Table 2).

**Table 2:** Unit-weighted and principal axis factor loadings of the acceptance and action questionnaire.

Acceptance and action questionnaire indicators	UWF loading	PAF loading
My painful experiences and memories make it difficult for me to live the life I would like to.	0.817	0.783
I am afraid of my feelings	0.791	0.744
I am worried about not being able to control my worries and feelings	0.829	0.797
My painful memories prevent me from living a fulfilling life	0.847	0.826
My emotions get in the way of how I want my life to be.	0.844	0.821
It seems that most people go through life better than I do	0.793	0.747
My worries get in the way of what I want to achieve.	0.817	0.780

The UCLA Loneliness Scale presented PAF loadings ranging from 0.678 to 0.913 and UWF loadings from 0.831 to 0.906. The eigenvalue was 1.971, and the CC between UWF and PAF was 0.996 (Table 3).

**Table 3:** Unit-weighted and principal axis factor loadings of the UCLA loneliness scale.

UCLA loneliness scale indicators	UWF loading	PAF loading
First, how often do you feel that you lack companionship	0.831	0.678
How often do you feel left out	0.906	0.913
How often do you feel isolated from others?	0.882	0.824

The UCLA Loneliness Scale featured a high reliability coefficient (α = 0.844) and exhibited PAF factor loadings ranging from 0.678 to 0.913. The analysis identified a single latent dimension (σ^2^ = 1.00; eigenvalue = 1.971). This measure included various items on the participant’s feeling of loneliness. The UWF factor scores were also sizeable in magnitude ranging from 0.831 to 0.906. Both factor structures featured high congruence (CC = 0.996).

The PHQ-4 yielded two subdimensions (anxiety and depression), with UWF loadings of 0.916 and 0.885, respectively. A higher-order factor structure was identified, with both domains loading onto a latent psychological distress dimension (*λ* = 0.912), which also showed good internal consistency (*α* = 0.798). This higher-order factor was significantly and negatively correlated with life satisfaction (Table 4).

**Table 4:** Unit-weighted loadings of patient health questionnaire of depression and anxiety.

Patient health questionnaire scale (PHQ)	UWF loading
PHQ anxiety domain indicators	
Feeling nervous, anxious, or on edge	0.916
Not being able to stop or control worrying	0.916
PHQ depression domain indicators	
Little interest or pleasure in doing things	0.885
Feeling down, depressed, or hopeless	0.885
PHQ higher-order depression and anxiety factor	
Anxiety domain	0.912
Depression domain	0.912
Higher-order mental health outcome factor	
Anxiety domain	0.836
Depression domain	0.868
Life satisfaction (R)	0.758

### 3.2 Bivariate correlations

As shown in Table 5, all correlations were statistically significant (*p* < 0.0001). Life satisfaction was negatively correlated with depression (r = −0.47), anxiety (r = −0.39), loneliness (r = −0.50), perceived stress (r = −0.52), and psychological inflexibility (r = −0.53). Psychological inflexibility was positively associated with perceived stress (r = 0.65) and loneliness (r = 0.63). Depression and anxiety were strongly correlated (r = 0.66), consistent with their clinical comorbidity.

**Table 5:** Bivariate correlation matrix among psychological inflexibility, region, sex, age, perceived stress, loneliness, depression, anxiety, and life satisfaction.

	LSQ2	DPQ	ANX	UCLA	PSS	AAQ
LSQ2	1.000	***	***	***	***	***
DPQ	−0.473	1.000	***	***	***	***
ANX	−0.394	0.664	1.000	***	***	***
UCLA	−0.504	0.492	0.437	1.000	***	***
PSS	−0.516	0.573	0.585	0.541	***	***
AAQ	−0.529	0.544	0.543	0.635	0.653	1.000

AAQ, psychological inflexibility; PSS, perceived stress; UCLA, loneliness; DPQANX, depression-anxiety; and LSQ2, life satisfaction. ***p < 0.0001.

### 3.3 Sequential canonical analysis (SEQCA): higher-order mental health outcome

The first SEQCA model evaluated the direct and indirect effects of psychological inflexibility, perceived stress, and loneliness on a higher-order mental health factor comprising depression, anxiety, and reversed life satisfaction. The overall model was statistically significant and explained 45% of the total variance (Table 6).

- Step 1: Psychological inflexibility significantly predicted perceived stress (sR = 0.65, *p* < 0.0001). Region and sex showed statistically significant but small effects. Age had no significant effect.
- Step 2: Perceived stress significantly predicted loneliness (sR = 0.54, *p* < 0.0001), and psychological inflexibility also showed a direct effect (sR = 0.33, *p* < 0.0001). Region and age were not significant predictors.
- Step 3: The higher-order mental health outcome was significantly predicted by loneliness (sR = 0.58), perceived stress (sR = 0.43), and psychological inflexibility (sR = 0.19). Region, sex, and age had statistically significant but minimal effects.

**Table 6:** Sequential canonical analysis evaluating the effects of psychological inflexibility, perceived stress, loneliness, on a depression-anxiety-life satisfaction factor.

Model	Effect size (E)	C.I.	F ratio	df1, df2	*p*-value
Overall (*V* = 0.600)	0.45	0.43,0.46	196.12	15/11775	<0.0001

	Effect size (sR)	C.I.	F ratio	df1, df2	*p*-value
Dv: PSS					
AAQ	0.65	0.63, 0.67	2971.84	1/3925	<0.0001
Region	0.03	0.01, 0.05	3.74	2/3925	0.0200
Sex	−0.09	−0.12, −0.06	61.42	1/3925	<0.0001
Age	0.01	−0.02, 0.05	1.51	1/3925	0.2200
Multiple R (Xs only)	0.66	0.65, 0.67	608.45	5/3925	<0.0001
Dv: UCLA					
Prior Y variable					
PSS	0.54	0.52, 0.56	1949.17	1/3924	<0.0001
X variables					
AAQ	0.33	0.31, 0.36	742.31	1/3924	<0.0001
Region	0.02	0.00, 0.04	1.02	2/3924	0.3600
Sex	0.06	0.03, 0.10	27.65	1/3924	<0.0001
Age	0.02	−0.01, 0.05	3.38	1/3924	0.0700
Multiple R (Xs only)	0.34	0.33, 0.35	155.07	5/3924	<0.0001
Dv: mental health outcome					
Prior Y variables					
UCLA	0.58	0.56, 0.60	3115.69	1/3923	<0.0001
PSS	0.43	0.41, 0.46	1727.24	1/3923	<0.0001
X variables					
AAQ	0.19	0.15, 0.22	314.83	1/3923	<0.0001
Region	0.11	0.10, 0.12	51.83	2/3923	<0.0001
Sex	0.02	−0.01, 0.05	4.88	1/3923	0.0300
Age	0.02	−0.01, 0.05	3.30	1/3923	0.0700
Multiple R (Xs only)	0.22	0.21, 0.23	85.34	5/3923	<0.0001

Although sex was statistically significant in several models (e.g., sR = −0.09 predicting perceived stress), the effect sizes were small, suggesting limited practical impact. Women reported slightly higher perceived stress and loneliness, consistent with prior research, but these differences accounted for less than 1% of the variance in outcomes.

**Figure 1:**
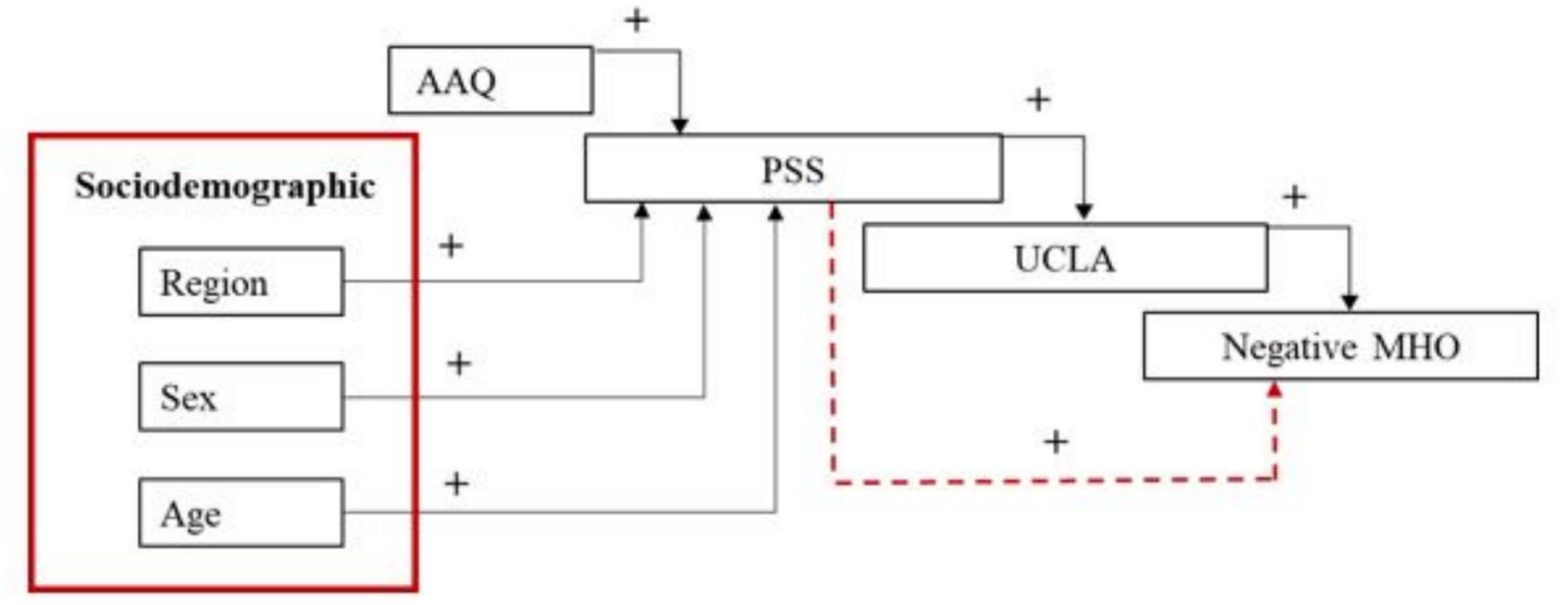
Sequential canonical analysis showing the predicted influence of psychological inflexibility (AAQ), region, sex, age, perceived stress (PSS), loneliness (UCLA), depression and anxiety (DPQANX) on life satisfaction. Dotted red lines indicate indirect effects; black lines indicate direct effects. A positive sign indicates a positive association; a negative sign indicates a negative association.

These sequential relationships are illustrated in Figure 1, which displays direct and indirect standardized estimates among variables.

### 3.4 Complementary SEQCA: life satisfaction as an independent outcome

A second Sequential Canonical Analysis (SEQCA) was conducted to examine life satisfaction as an independent outcome, rather than as part of a higher-order latent mental health factor. This complementary model allowed for a more nuanced exploration of the specific pathways through which psychological inflexibility, perceived stress, and loneliness influence subjective well-being.

The model was statistically significant and explained 35% of the variance in life satisfaction (see Table 7).

**Table 7:** Sequential canonical analysis evaluating the influence of psychological inflexibility, region, sex, age, perceived stress, loneliness, depression, and anxiety on life satisfaction.

Model	Effect size (E)	C.I.	F ratio	df1, df2	*p*-value
Overall (*V* = 0.623)	0.35	0.33,0.37	111.73	25/19625	<0.0001

	Effect size (sR)	C.I.	F ratio	df1, df2	*p*-value
Dv: ANX					
Prior Y variables					
UCLA	0.44	0.41, 0.46	1220.2	1/3923	<0.0001
PSS	0.41	0.39, 0.44	1094.66	1/3923	<0.0001
X variables					
AAQ	0.14	0.11, 0.17	128.19	1/3923	<0.0001
Region	0.04	0.02, 0.06	4.76	2/3923	0.0090
Sex	−0.04	−0.07,-0.01	10.78	1/3923	0.0010
Age	−0.02	−0.05, 0.01	2.41	1/3923	0.1200
Multiple R (Xs only)	0.15	0.14, 0.16	30.18	5/3923	<0.0001
Dv: DPQ					
Prior Y variables					
ANX	0.66	0.65, 0.68	3642.3	1/3922	<0.0001
UCLA	0.22	0.19, 0.25	417.82	1/3922	<0.0001
PSS	0.15	0.12, 0.18	189.52	1/3922	<0.0001
X variables					
AAQ	0.06	0.03, 0.09	33.59	1/3922	<0.0001
Region	0.04	0.03, 0.05	7.99	2/3922	0.0003
Sex	0.07	0.04, 0.10	41.65	1/3922	<0.0001
Age	0.01	−0.02, 0.04	0.76	1/3922	0.3800
Multiple R (Xs only)	0.11	0.09, 0.12	18.4	5/3922	<0.0001
Dv: LSQ2					
Prior Y variables					
DPQ	−0.47	−0.50,-0.45	1453.68	1/3921	<0.0001
ANX	−0.11	−0.14,-0.08	73.89	1/3921	<0.0001
UCLA	−0.30	−0.33,-0.27	574.41	1/3921	<0.0001
PSS	−0.19	−0.22,-0.16	238.36	1/3921	<0.0001
X variables					
AAQ	−0.11	−0.14,-0.07	72.94	1/3921	<0.0001
Region	0.14	0.13, 0.15	60.34	2/3921	<0.0001
Sex	−0.04	−0.07,-0.01	9.64	1/3921	0.0020
Age	−0.06	−0.09,-0.03	23.69	1/3921	<0.0001
Multiple R (Xs only)	0.19	0.18, 0.20	45.39	5/3921	<0.0001

**Figure 2:**
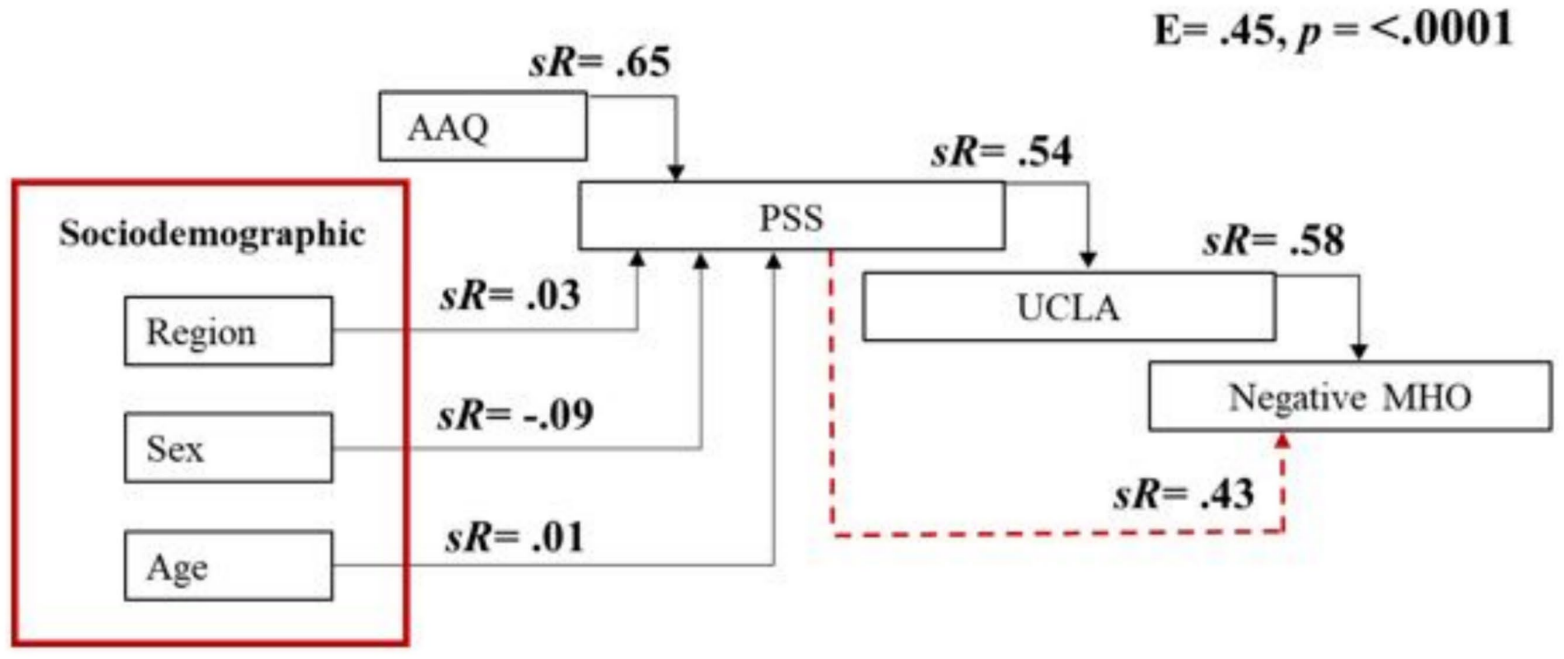
Sequential canonical analysis evaluating the influence of psychological inflexibility (AAQ), region, sex, age, perceived stress (PSS), loneliness (UCLA), and mental health outcomes (MHO) on life satisfaction (LSQ2). Red lines represent indirect effects; black lines represent direct effects.

As shown in Figure 2, the analytic sequence began with psychological inflexibility, region, sex, and age; followed by perceived stress and loneliness; and finally depression and anxiety as mediators of life satisfaction.

Specifically: (1) Anxiety scores were positively predicted by loneliness (sR = 0.44, *p* < 0.0001), perceived stress (sR = 0.41, *p* < 0.0001), and psychological inflexibility (sR = 0.14, *p* < 0.0001). Region and sex had statistically significant but small effects; age was not significant. (2) Depression scores were positively predicted by anxiety (sR = 0.66, *p* < 0.0001), loneliness (sR = 0.22, *p* < 0.0001), perceived stress (sR = 0.15, *p* < 0.0001), and psychological inflexibility (sR = 0.06, *p* < 0.0001). Again, region and sex contributed small but significant effects, while age remained non-significant. (3) Life satisfaction was negatively predicted by depression (sR = −0.47, *p* < 0.0001), anxiety (sR = −0.11, *p* < 0.0001), loneliness (sR = −0.30, *p* < 0.0001), perceived stress (sR = −0.19, *p* < 0.0001), and psychological inflexibility (sR = −0.11, *p* < 0.0001). Although region, sex, and age contributed statistically to the model, their effect sizes were negligible.

**Figure 3:**
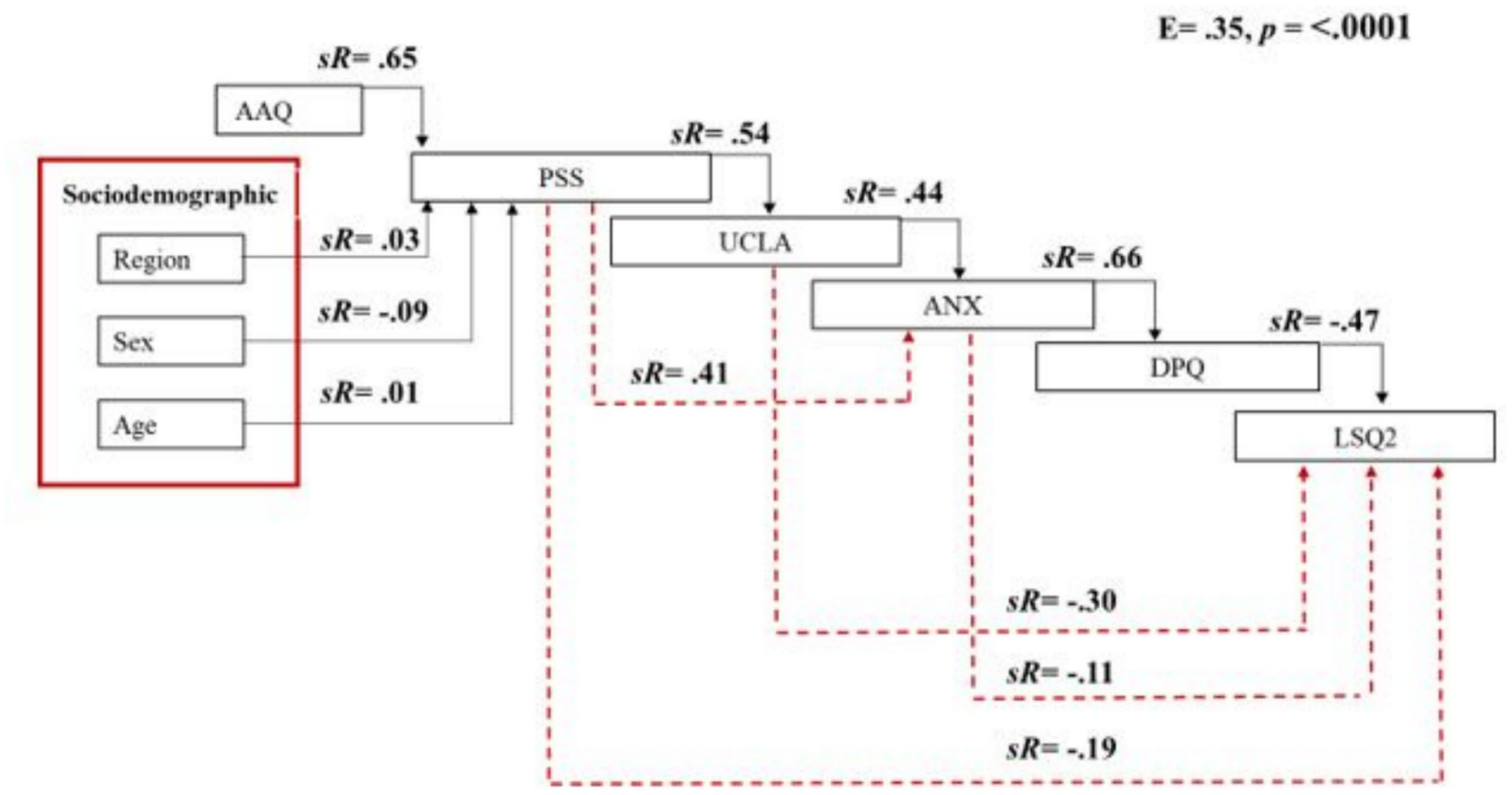
Sequential canonical analysis evaluating the influence of psychological inflexibility (AAQ), region, sex, age, perceived stress (PSS), loneliness (UCLA), depression-anxiety (DPQANX) on life satisfaction (LSQ2). Red lines represent indirect effects; black lines represent direct effects.

This complementary model underscores the distinct contribution of each psychological variable to life satisfaction, while also reflecting the cumulative and mediating roles played by anxiety and depression. Figure 3 provides a visual summary of this model, illustrating the sequential effects and standardized estimates for each path.

Finally, although sociodemographic factors such as sex and region contributed significantly at several stages of the model, their effect sizes were consistently small. Age did not exert a significant influence on any of the outcomes. These results further underscore the dominant role of transdiagnostic psychological factors over demographic characteristics in shaping students’ mental health and well-being.

## 4 Discussion

This study examined the transdiagnostic pathways through which psychological inflexibility, perceived stress, and loneliness contribute to mental health outcomes among university students. Using Sequential Canonical Analysis, we identified both direct and indirect effects linking these variables to anxiety, depression, and life satisfaction. The findings provide robust empirical support for integrated models of psychological vulnerability, while also offering actionable insights for public mental health strategies in higher education settings.

It is important to note that the cross-sectional nature of this study precludes causal inference, and all measures were self-reported. These limitations should be considered when interpreting the findings and their implications.

### 4.1 Main findings and theoretical implications

Consistent with prior literature (10, 33), psychological inflexibility emerged as a central predictor, exerting its effects both directly and indirectly via stress and loneliness. This finding supports the conceptualization of inflexibility as a transdiagnostic process that amplifies emotional reactivity and impairs adaptive coping (32). Our data further suggest that psychological inflexibility not only intensifies perceived stress, but also contributes to feelings of loneliness—an often overlooked association that warrants greater attention in research and intervention design.

Perceived stress served as a key mediating variable, linking inflexibility to both loneliness and adverse mental health outcomes. This aligns with the stress generation hypothesis (31), which posits that cognitive-emotional traits increase the likelihood of encountering or interpreting life events as stressful. In our sample, stress was not merely an outcome, but an active transmitter of psychological risk.

Perhaps most strikingly, loneliness demonstrated the strongest association with the composite mental health outcome, as well as with life satisfaction when examined independently. These results reinforce the growing recognition of loneliness as a form of chronic social stress with serious psychological and physiological consequences (15, 23). That loneliness accounted for more variance in depression and anxiety than did stress or inflexibility underscores its relevance as a public mental health priority in young adults.

Similar studies in European and North American university samples have also found that loneliness exerts a stronger influence on depression and anxiety than other stress-related constructs, underscoring its role as a transdiagnostic process across cultural contexts (2, 7). Our findings add to this literature by demonstrating that these patterns are also evident in an understudied Latin American population.

Drawing on Rose’s (34) distinction between the causes of cases and the causes of incidence, our findings suggest that while psychological inflexibility may reflect individual susceptibility, loneliness and stress may constitute contextual exposures that elevate risk across the population. In this framework, inflexibility functions as a vulnerability marker, whereas loneliness—exacerbated by structural and cultural shifts in student life—may serve as a population-level determinant of incidence. This perspective argues for shifting the intervention focus beyond individual traits toward modifiable social conditions.

### 4.2 Clinical and public health implications

The results have meaningful implications for both individual-level interventions and broader health promotion strategies. From a clinical standpoint, the prominent role of psychological inflexibility supports the continued development of acceptance- and mindfulness-based approaches, such as Acceptance and Commitment Therapy (ACT), for university populations (24, 25). At the same time, our findings highlight the importance of integrating modules on stress appraisal and social connection into psychological services, particularly in early intervention contexts.

At the public health level, the magnitude of the association between loneliness and poor mental health suggests that targeting social integration should be a key component of university mental health promotion. Interventions that foster peer belonging, community building, and opportunities for meaningful engagement may reduce population-level incidence of psychological distress (26, 27). These findings also support calls to frame loneliness as a public health issue, particularly in youth (28, 29), and to design universal preventive interventions rather than focusing solely on high-risk individuals (34). Specific interventions could include campus-based acceptance and commitment therapy workshops to enhance psychological flexibility, structured peer mentoring programs to foster belonging, and universal screening for loneliness during routine student health assessments. Implementing such interventions requires institutional commitment and resources.

Importantly, our models explained a substantial proportion of variance: 45% for the higher-order mental health factor and 35% for life satisfaction. These values are noteworthy given the complexity and multifactorial nature of mental health outcomes and underscore the explanatory power of transdiagnostic frameworks in applied settings.

### 4.3 Limitations and future directions

The use of the terms “predict,” “predictor,” and “prediction” throughout this manuscript refers solely to statistical prediction within the regression models employed. No causal inference can be made due to the cross-sectional study design. Additionally, several limitations should be acknowledged. First, although SEQCA allows for modeling temporal order based on theory, the cross-sectional design ultimately precludes definitive causal conclusions, and longitudinal studies are needed to confirm directional relationships. Second, all measures relied on self-report instruments, which may introduce shared method variance or social desirability bias. Third, while the large and diverse sample enhances generalizability within Ecuador, cultural and contextual factors may limit extrapolation to other regions or educational systems. Cultural factors in Ecuador, such as strong family ties and collectivist orientations, may both buffer and exacerbate loneliness in university contexts. For example, moving away from home for study may entail heightened separation distress. Caution should be exercised in extrapolating these findings to countries with different social structures. Future cross-cultural studies are recommended. Finally, future research should explore alternative analytic approaches (e.g., structural equation modeling with latent interactions) and examine potential non-linear or threshold effects.

## 5 Conclusion

This study demonstrates that psychological inflexibility, perceived stress, and loneliness operate as interconnected transdiagnostic processes with significant implications for mental health and life satisfaction in university students. The findings support the application of integrated frameworks in both clinical practice and public health and highlight the need to move beyond individual symptomatology toward contextual and relational determinants of well-being. In line with Rose’s (34) vision, population-level strategies that address the social architecture of student life—particularly social isolation—may offer the most promising path toward sustainable mental health promotion.

## Data Availability

The raw data supporting the conclusions of this article will be made available by the authors, without undue reservation.
